# Improving Wellbeing in At‐Risk Familial frontotemporal dementia (IWARF): a feasibility study of a blended digital/human online intervention

**DOI:** 10.1002/alz.089063

**Published:** 2025-01-09

**Authors:** Emma Harding, Oliver S Hayes, Emilie V Brotherhood, Caroline V Greaves, Sophie E Goldsmith, Jonathan D. Rohrer, Joshua Stott

**Affiliations:** ^1^ Dementia Research Centre, UCL Queen Square Institute of Neurology, University College London, London United Kingdom; ^2^ Department of Clinical, Educational, and Health Psychology, Division of Psychology and Language Sciences, University College London, London United Kingdom

## Abstract

**Background:**

Familial frontotemporal dementia is an autosomal dominant heritable form of frontotemporal dementia, a form of dementia characterised by changes in personality, behaviour and communication which typically onsets in mid‐life. Children of an affected parent are at 50% risk of inheriting the responsible genetic mutation and developing frontotemporal dementia themselves. Individuals living at‐risk have high psychological morbidity, for example they report struggling with guilt and anxiety about risk to themselves and their children, decisions about whether to get tested, uncertainty about onset of symptoms, and see their risk as a barrier in life. Despite this high psychological morbidity there are no tailored interventions available to support those living at‐risk of familial frontotemporal dementia.

**Methods:**

Guided by Medical Research Council frameworks we have co‐developed a brief blended Acceptance and Commitment Therapy‐informed person/digital intervention to support individuals at‐risk of familial frontotemporal dementia. The intervention was developed in consultation with experts‐by‐experience and clinical neurology, psychology and genetic counselling experts. It is designed to be self‐paced over 6‐8 weeks, and involves a number of online modules including informational content, videos and independent activities, interspersed with three one‐hour check‐in sessions with a facilitator. We are conducting a mixed‐methods feasibility study (n = 10), with participants with a range of genetic statuses (known mutation carrier, known non‐carrier, unknown). Participants will complete pre‐ and post‐ measures of mood, adaptation to genetic information and psychological flexibility and a qualitative interview to capture their experience of the intervention and its impact on wellbeing.

**Results:**

We will report on adherence, attrition and acceptability of the intervention, any identified barriers and facilitators to engaging with the intervention, and plans for adaptation and refinement.

**Conclusion:**

Findings will inform the refinement of the intervention and determine primary and secondary outcome measures for a full randomised controlled‐trial starting in Spring 2024 to test the efficacy of this first‐of‐its‐kind blended digital/human online intervention to improve wellbeing for those living at‐risk of familial frontotemporal dementia. Findings will also inform adaptation to other autosomal dominant conditions (e.g. familial Alzheimer’s disease), in which there are some disease‐specific differences in symptomatology alongside similar unmet psychological needs for those living at‐risk.

